# Invasive Insect Species: Global Challenges, Strategies & Opportunities

**DOI:** 10.3389/finsc.2021.650520

**Published:** 2021-04-08

**Authors:** Robert C. Venette, William D. Hutchison

**Affiliations:** ^1^Forest Service, US Department of Agriculture, St. Paul, MN, United States; ^2^Minnesota Invasive Terrestrial Plants and Pests Center, University of Minnesota, St. Paul, MN, United States; ^3^Department of Entomology, University of Minnesota, St. Paul, MN, United States

**Keywords:** prediction, prevention, early detection, rapid response, integrated pest management, grand challenges, wicked problem, global development

Alien arthropods threaten human health, jeopardize food supplies, endanger valued species, risk economic losses, and disrupt ecosystem functions, at least some of the time. The accelerating pace of commercial and social globalization creates unprecedented opportunities for the movement of species to new areas of the world. As arthropods are dispersed beyond their native ranges, they escape population-regulating predators, parasitoids, and pathogens and encounter naïve host plants and animals. In principle, the disruption of coevolutionary, antagonistic interactions presents an invading species with an opportunity to dominate and transform invaded areas, as evidenced, by grape phylloxera, *Daktulosphaira vitifoliae*, in Europe in the mid 1800's and emerald ash borer, *Agrilus planipennis*, in North America and Europe in the early 2000's. Nevertheless, invasion by severe pests remains an improbable happening, estimated at approximately one “success” for every 1,000 attempts (1). Thus, biological invasions represent low-probability, high-consequence events, predicaments to those who study or manage alien invasive pests.

Invasions involving arthropods are perhaps the most pervasive, underappreciated component of grand challenges to global development and prosperity, particularly to human health, nutritious food, clean water, resilient environments, and sustainable economies. Direct effects on food or the environment can be readily appreciated, as when fall armyworm, *Spodoptera frugiperda*, consumes maize in Africa (2), invasive flies, *Philornis downsi*, attack Darwin's finches in the Galapagos (3, 4), or red turpentine beetle, *Dendroctonus valens*, kills expanses of pine in China (5). Indirect effects may be more cryptic, as when brown marmorated stink bug, *Halyomorpha halys*, vectors aflatoxin-producing fungi in the USA (6), spittlebugs spread the non-native bacterium *Xylella fastidiosa* (7), or Asian tiger mosquito, *Aedes albopictus*, transmits dengue viruses in South America (8). Further indirect effects to health, water, and the environment can arise through responses to arthropod invasions, especially if intensive pesticide use becomes necessary (9). Perhaps least appreciated are the barriers invasive arthropods present to economic advancement in developing nations when demonstrable phytosanitary concerns preclude or curtail international trade in plant-based commodities from those nations to global markets. Thus, solutions to the problem of arthropod invasions are needed to achieve diverse, humanitarian goals.

We are excited about this opportunity to support a new venue for publishing research and conceptual ideas, with a focus on invasive arthropods. C.S. Elton (10), the founder of modern invasion biology, provided numerous insect examples among others to highlight commonalities of the invasion process. Recent reviews feature promising areas for invasive species research in general and emphasize the value of studying across spatiotemporal scales, collaborating with international partners, engaging the public, and considering interactions with climate change or other environmental stressors (11, 12). We believe a renewed, clear emphasis on arthropod invasions will accelerate progress for entomologists and invasion biologists alike. The purpose of this overview is to highlight current challenges and offer a vision for moving the field of invasive arthropod species ecology and management forward via insightful articles that not only present new research but also challenge long-held theory and practice relevant to invasive species biology, ecology and management. We are motivated not by the novelty, but by the consequences, of new species in new environments. We seek answers that are well-grounded in theory and rigorously tested in practice.

## Grand Challenges for Invasive Insect Science

Invasion biology is inherently an applied discipline, and the core questions remain: (1) which species are most likely to invade; (2) where are invasions most likely to be successful; and (3) how can this information be used “to best advantage” to manage biological invasions (13)? As invasions progress through the phases of arrival, establishment, and spread, management strategies change from prediction and prevention, to early detection and rapid response, to mitigation and management. Bioeconomic analyses generally confirm that efforts to prevent or contain invasions are more cost-effective than efforts to mitigate damage or restore ecosystems after invasion has occurred (14, 15).

Alien invasive species are what sociologists call a “wicked” problem with multiple causes (e.g., international trade, phytosanitary breakdowns, climate change, public unawareness, etc.), each defying a simple, outright solution (16, 17). Researchers can help tame the invasive species problem by addressing goals with strong public support and tangible metrics of success. The value of a proposed solution (e.g., new sampling plan or control technology) should be transparent and highlight the costs and benefits relative to current practice.

### Prediction and Prevention

Prediction and prevention remain the most lauded approach to address alien invasive species. The intent seems simple enough: forecast which species or pathways pose unacceptable risks and keep them from arriving in an area of concern. This strategy requires a clear benchmark of success. As the number of introduced arthropod species continues to increase at a steady rate, each new incursion might be viewed as evidence of a biosecurity failure (11). However, global trade has increased more rapidly than species arrivals, a pattern that suggests biosecurity efforts have been somewhat effective (17) but is far from a clear measure of achievement (18).

We see promise in future systems integration to forecast threats and prevent introductions. We remain optimistic that advances in horizon scanning and real-time global pest surveillance can provide more dynamic representations of encroaching threats (19, 20). We see opportunities in systems approaches to keep invasive alien insects out of supply chains for goods destined for foreign markets. Advances in commodity disinfestation technologies remain needed, with alternatives to methyl bromide still a priority (21). In addition, more sophisticated tools, perhaps based on environmental DNA or volatile chemical profiles, are needed to detect insects *en route* (22, 23).

Spatially-explicit pest risk assessment will remain a cornerstone of biosecurity efforts both to inform the permissibility of a good for import, to structure surveillance programs, and to conduct post incursion responses should a species arrive within an endangered area (24). These assessments generally describe where a species is most likely to invade and/or cause harm, often focusing on conditions needed for pest establishment. However, approaches for developing these assessments are needed that are reliable, scalable, and affordable. The conceptual challenge of developing models that are reliably transferable to new space and time is becoming increasingly recognized, particularly for machine learning and other statistical models (25). Process-based models have appeal, but extensive data requirements make the approach prohibitive for application to the hundreds, if not thousands, of species of concern (26). Advances in the democratization of data collection through apps and open access databases improve capacity to capture changes in distribution, abundance, and phenology of insect populations. Developments in phylogeography will provide a more detailed accounting of invasions, identify invasive phenotypes, and more explicitly account for genotype x environment interactions to improve the rigor of forecasts (27–29).

### Early Detection and Rapid Response

Should prediction and prevention efforts fail, the next best alternative is to locate nascent populations of high-risk species early and eradicate or contain them quickly. Despite the global development of methods for early detection of invasive insects, initial observations of alien species in both agricultural and forest systems are still too often fortuitous (17). Research to improve this strategy is needed to address where/when/how to look for new invasive species and how to confirm their identity if encountered.

Pest risk maps highlight areas where species might invade or cause harm, and as such, provide useful direction about areas to consider for early-detection surveys. The endangered area may be too large to survey effectively with available resources. We believe more research is needed to quantify the probability of detecting low densities of invasive arthropods with a particular sampling scheme (30). A “risk-based surveillance” approach has been developed for plant pathogens that targets sampling effort where most preferred and valuable host plants occur (31). The cost of invasive species detection can therefore be reduced, yet more research is needed to examine this approach for invasive insects. Yemshanov et al. (32, 33) have taken spatial optimization of sampling effort to a new level, but the methods can be computationally intensive to apply.

Within endangered areas, the next major challenge becomes how to sample for the early detection of targeted species. Sentinel plots of appropriate plant species can be used effectively to detect species or suites of invasive arthropods that share a common host, for example, maize (34) or maples (35). The deployment of traps, particularly when appropriate semio-chemical lures are available, remains a preferred monitoring option because traps are more convenient and cost-effective than visual inspections. Nevertheless, visual inspections may be unavoidable if lures are unavailable or traps become impractical. Citizen science to assist with trap monitoring or visual inspection has been embraced for the early detection of spotted-wing drosophila, *Drosophila suzukii*, in the United States (36) and the multicolored Asian lady beetle, *Harmonia axyridis*, in Europe (37), for example.

Recent advances in genomics, bolstered by traditional taxonomic expertise, offer a path to rapid confirmation of species identity. An additional advantage of genomic approaches is that various strains, biotypes or haplotypes can be distinguished within a species. Specific haplotype data can thereby reflect more precisely the geographic origins of an invasive population, as demonstrated for walnut twig beetle, *Pityophthorus juglandis* (38), *H. halys* (39), and *S. frugiperda* (40). With accurate knowledge of pest origins, specific countries can be targeted early in the search for natural enemies, usually species-specific parasitoids, for subsequent safety and efficacy evaluation and release for biological control within the invaded countries (41).

### Management and Mitigation

Following early establishment and spread of invasive arthropods, immediate investment in research and development to support integrated pest management (IPM) solutions continues to be a valid paradigm. IPM is “an ecosystem-based strategy that focuses on long-term prevention of pests or their damage through a combination of techniques such as biological control, habitat manipulation, modification of cultural practices, and use of resistant varieties. Pesticides are used only after monitoring indicates they are needed according to established guidelines, and treatments are made with the goal of removing only the target organism. Pest control materials are selected and applied in a manner that minimizes risks to human health, beneficial and non-target organisms, and the environment” (42). Area-wide pest management extends these decision-making principles beyond individual farms or landholdings.

Ironically, for many cropping systems, IPM is often in place for endemic pests when invasive pests arrive. Some arrivals disrupt existing IPM programs where biological control and/or reduced-risk insecticides are being used; *H. halys* in U.S. apple production is a prime example, where >$37 million was lost due to damage and increased insecticide application costs (43). As with endemic pests, the IPM response to invasive species typically requires both near-term and long-term strategies. To protect grower investments, the initial focus is often insecticidal control, with simultaneous commitments to fund research on biological control, pest-resistant varieties, cultural controls (44), physical exclusions (45), and “attract and kill” trap-based systems based on insect behavior (46). Breeding for resistance to invasive pests is increasingly important for trees and perennial crops. Transgenic insecticidal plants (47) or genetic biocontrol agents (48, 49), as through gene-drives, are novel technologies that could supplement or supplant IPM programs, pending requisite regulatory approvals.

## Conclusions

We look forward to hosting the first journal with a section fully devoted to invasive arthropod biology, ecology and management. Broadly, we envisage papers focused on invasive arthropods of current or future significance to human health, agriculture, forestry, or natural ecosystems. We welcome comprehensive research, review, and perspective articles that address impacts of invasive arthropods on food security, global trade, forest and ecosystem health, biodiversity, crop protection, public health and recreation, and policy. Specifically for *Frontiers in Insect Science*, our section encourages papers in population/community and landscape ecology, population genetics and evolutionary biology, pest risk assessment and mapping, the intersection of invasive species spread and global climate change, novel methods for early detection, biological control, genetic biocontrol, area-wide integrated pest management, and economic and environmental impacts. We look forward to the journey!

## Author Contributions

RV and WH were the sole contributors to this manuscript. Both authors contributed to the article and approved the submitted version.

## Conflict of Interest

The authors declare that the research was conducted in the absence of any commercial or financial relationships that could be construed as a potential conflict of interest.
